# Singlet Oxygen in Antimicrobial Photodynamic Therapy: Photosensitizer-Dependent Production and Decay in *E. coli*

**DOI:** 10.3390/molecules18032712

**Published:** 2013-02-28

**Authors:** Xavier Ragàs, Xin He, Montserrat Agut, Mónica Roxo-Rosa, António Rocha Gonsalves, Arménio C. Serra, Santi Nonell

**Affiliations:** 1 Grup d’Enginyeria Molecular, Institut Químic de Sarrià, Universitat Ramon Llull, Barcelona E-08017, Spain; E-Mails: xavierragasa@iqs.url.edu (X.R.); xin.x.he@gmail.com (X.H.); montserrat.agut@iqs.url.edu (M.A.); 2 Center for Biodiversity, Functional & Integrative Genomics, Faculty of Sciences, University of Lisbon, Lisbon 1749-016, Portugal; 3 Department of Genetics, National Institute of Health Dr. Ricardo Jorge, Lisbon 1649-016, Portugal; E-Mail: roxorosa@hotmail.com; 4 Departamento de Quimica, Universidade de Coimbra, Coimbra P-3049535, Portugal; E-Mails: arg@qui.uc.pt (A.R.G.); armenio.serra@gmail.com (A.C.S.)

**Keywords:** antimicrobial photodynamic therapy, cationic photosensitizers, *E. coli*, kinetics, photodynamic inactivation, singlet oxygen, time-resolved near-IR spectroscopy

## Abstract

Several families of photosensitizers are currently being scrutinized for antimicrobial photodynamic therapy applications. Differences in physical and photochemical properties can lead to different localization patterns as well as differences in singlet oxygen production and decay when the photosensitizers are taken up by bacterial cells. We have examined the production and fate of singlet oxygen in *Escherichia coli* upon photosensitization with three structurally-different cationic photosensitizers, namely New Methylene Blue N (NMB), a member of the phenothiazine family, ACS268, a hydrophobic porphyrin with a single cationic alkyl chain, and zinc(II)-tetramethyltetrapyridinoporphyrazinium salt, a phthalocyanine-like photosensitizer with four positive charges on the macrocycle core. The kinetics of singlet oxygen production and decay indicate different localization for the three photosensitizers, whereby NMB appears to localize in an aqueous-like microenvironment, whereas ACS268 localizes in an oxygen-shielded site, highly reactive towards singlet oxygen. The tetracationic zinc(II) tetrapyridinoporphyrazine is extensively aggregated in the bacteria and fails to produce any detectable singlet oxygen.

## 1. Introduction

Antimicrobial photodynamic therapy (APDT) [1] has been shown as a viable alternative to the use of antibiotics against microbial pathogens, even to those that have developed resistance [2,3]. Briefly, the combination of a drug, usually referred to as the photosensitizer (PS), light and molecular oxygen leads to the production of reactive oxygen species (ROS), via electron or energy transfer (type I or type II reactions, respectively) from the triplet excited-state of the PS to molecular oxygen [4].

Many different PSs have been used over the years as antimicrobial agents in APDT [5,6]. Nowadays, the most effective ones are cationic at physiological pH, e.g., phenothiazines, porphyrins and phthalocyanines [7,8,9]. These PSs exhibit high efficiency against microbial cells, especially against Gram-negative bacteria, due to the negative net charge found in the microbial cell walls of the latter [10,11,12].

In a previous study we reported how, by means of a combination of spectroscopic and time-resolved photophysical techniques, it is possible to understand where the PS localizes and how singlet oxygen (^1^O_2_), one of the reactive oxygen species that plays a major role in APDT [13], is produced and reacts with oxidizable biomolecules in its nearest environment. In that case, tetra-*N*-methylpyridylporphyrin (TMPyP) was used as a model, demonstrating a double localization both inside and outside the cell, and the ability of ^1^O_2_ to cross the cell wall and react with the external aqueous media [14].

**Figure 1:**
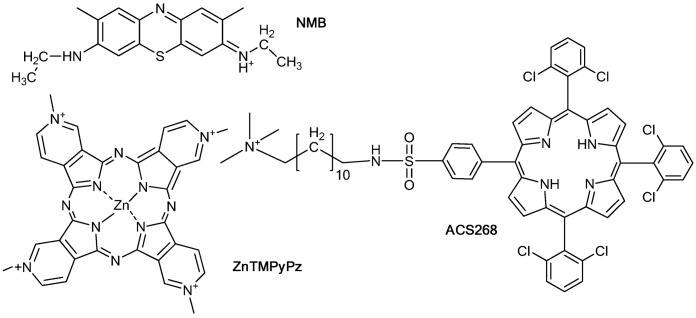
Chemical structures of the photosensitizers used in the study: NMB: New Methylene Blue N; ZnTMPyPz: zinc tetramethyltetrapyridino[3,4-b:39,49-g:30,40-l:3-,4-q]porphyrazinium salt; ACS268: 5,10,15-tris(2,6-dichlorophenyl)-20-[*N*-(12-trimethyl ammonium chloride)-dodecyl-*p*-sulphonamidophenyl]porphyrin.

In this paper we report the differences in the kinetics of ^1^O_2_ production and decay in *Escherichia coli* upon photosensitization with three different cationic PSs chosen as representative members of the aforementioned families, *i.e.*, the phenothiazine New Methylene Blue N (NMB), a hydrophobic porphyrin with a cationic alkyl chain (ACS268), and a tetracationic zinc(II) pyridine-porphyrazine, namely zinc(II) tetramethyltetrapyridino[3,4-b:39,49-g:30,40-l:3-,4-q]porphyrazinium salt (ZnTMPyPz) (Figure 1). NMB and ZnTMPyPz are efficient antimicrobial photodynamic agents *in vitro* [15,16,17,18], whereas ACS268 is inactive against *E. coli*, although it shows some activity against *S. aureus*.

Aside from their cationic charges, each PS possesses different physical, chemical, and photophysical properties [6]. These variations should likely lead to a different PS localization and therefore, to a different microenvironment. It may therefore be insightful to study the details of ^1^O_2_ photosensitization by different PSs to better understand the structure-activity relationships of different families of PS.

## 2. Results and Discussion

### 2.1. PSs Binding to E. coli

The uptake of NMB and ACS268 was studied by treating the cells with NaOH 0.1 M/1% sodium dodecyl sulphate (SDS) in order to extract the cell-bound PSs and analysing the cell lysates by fluorescence spectroscopy. Bulk concentrations of 10 µM and 7.5 µM were used, respectively. While the uptake of ACS268 reaches a plateau value after only 1 h, the uptake of NMB requires *ca.* 20 h for completion (see Supplementary Information). For ZnTMPyPz we adopted the uptake conditions described by Dupouy *et al.* [15], namely 30 min contact time at 37 °C and 10 µM bulk concentration.

It was critical in our experiments to eliminate any PS molecule from the external aqueous buffer in order to ensure that all the spectroscopic data was related to cell-bound molecules. Therefore, to unmistakably assign the spectroscopic data, the external aqueous solutions were routinely tested before and after the *in vitro* measurements, providing no significant signal in all cases.

**Figure 2:**
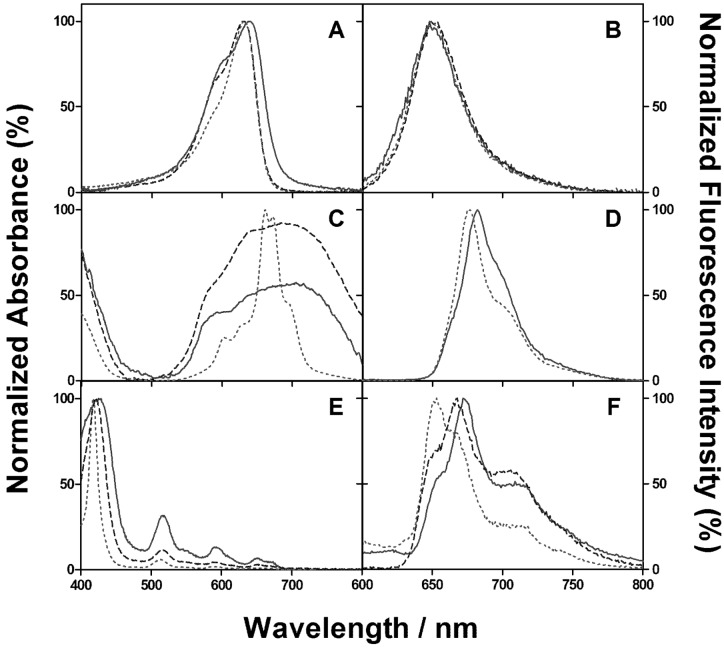
(**A**,**C**,**E**) Absorption and (**B**,**D**,**F**) fluorescence spectra of: (**A**,**B**) New Methylene Blue N in *E. coli* (solid line), PBS (dashed line), 1% SDS in 0.1 M NaOH (dotted line). (**C**,**D**) ZnTMPyPz in *E. coli* (solid line), PBS (dashed line), DMF (dotted line). (**E**,**F**) ACS268 in *E. coli* (solid line), PBS (dashed line), DMF (dotted line).

The absorption spectrum of NMB bound to *E. coli* showed a red-shift of 8 nm relative to that in the buffer (Figure 2A), as well as a slight change in the relative amplitudes of the band and the shoulder. However, no significant change was observed in the fluorescence emission spectrum (Figure 2B). For ZnTMPyPz, the absorption spectra both in *E. coli* and buffer showed a similar structureless broad-band distinct from the spectrum obtained in DMF, where it is in a monomeric form (Figure 2C). A 5 nm red shift was observed in the fluorescence spectrum relative to that in DMF (Figure 2D). For ACS268, the absorption spectrum in *E. coli* showed a 7 nm red shift relative to that in buffer (Figure 2E). The fluorescence spectra in *E. coli* and buffer showed similar structures but with a 5 nm red shift (Figure 2F).

Time-resolved fluorescence measurements provided additional insight on the uptake of the PSs (see Electronic Supplementary Information). The fluorescence of NMB at 657 nm decayed with first-order kinetics with a lifetime of 0.5 ± 0.1 ns both in *E. coli* and in buffer. The fluorescence decay of ZnTMPyPZz in *E. coli* at 675 was also fitted with a single exponential function with a lifetime of 2.8 ± 0.5 ns. An additional exponential term was necessary to fit the data in buffer, namely with a lifetime of 0.5 ± 0.1 ns. Time-resolved fluorescence of ACS268 was inconclusive as the kinetics in solution was too complex to be fitted by any standard model.

### 2.2. Singlet Oxygen Kinetics in E. coli Cells with NMB as PS

**Figure 3:**
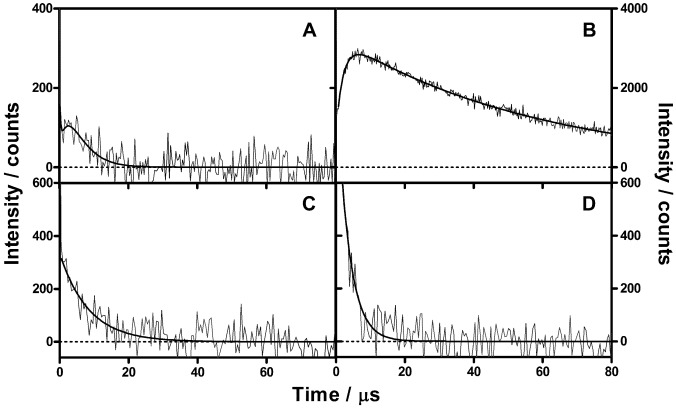
Singlet oxygen phosphorescence at 1270 nm using NMB as photosensitizer in (**A**) *E. coli*/PBS in air-saturated atmosphere, (**B**) *E. coli*/D-PBS in air-saturated atmosphere, (**C**) *E. coli*/D-PBS + 0.75 mM BSA in air-saturated atmosphere. (**D**) *E. coli*/D-PBS + 0.75 mM BSA in oxygen-saturated atmosphere.

Pulsed-laser irradiation of NMB-loaded *E. coli* cells (30 min contact time; 10 μM NMB bulk concentration) suspended in buffer at pH 7.4 produced clear time-resolved ^1^O_2_ phosphorescence signals at 1,270 nm (Figure 3). The contact time between the NMB solution and the bacteria had no effect on the ^1^O_2_ luminescence kinetics. In PBS, the signals grew with a time constant of τ_1_^PBS^ = 2.5 ± 1 μs, and decayed monoexponentially with a lifetime τ_2_^PBS^ = 3.7 ± 1 μs. In deuterated-PBS (D-PBS), the signal rise remained unchanged with a lifetime τ_1_^D−PBS^ = 2.5 ± 1 μs, but it decayed more slowly, with a time constant of τ_2_^D−PBS^ = 65 ± 2 μs. Addition of 0.75 mM bovine serum albumin (BSA), a ^1^O_2_ quencher that is not able to cross the cell wall due to its size [19], clearly modified the signal: the rise of the phosphorescence was now complete within the time resolution of our set-up, and the decay was also faster with lifetime τ_1_^BSA^ = 7.0 ± 1 μs. Saturation of this suspension with oxygen led to a further decrease of the decay lifetime to τ_1_^BSA,O2^ = 3.5 ± 1 μs. Finally, measurements in *E. coli* spheroplasts, *i.e.*, bacteria from which the cell wall had been almost completely removed, led to a 4-fold decrease in the ^1^O_2_ phosphorescence signal intensity, but with the same kinetics as in intact cells (see Supplementary Information).

### 2.3. Singlet Oxygen Kinetics in E. coli Cells with ZnTMPyPz as PS

Although ZnTMPyPz is a potent ^1^O_2_ photosensitizer in solution [20], irradiation of ZnTMPyPz-loaded *E. coli* cells suspended in D-PBS did not produce any clear time-resolved ^1^O_2_ phosphorescence signal at 1,270 nm (see Supplementary Information). Neither a higher contact time of ZnTMPyPz (3 h), nor a higher acquisition time of the ^1^O_2_ phosphorescence signal (12 million laser pulses) produced any detectable signal.

### 2.4. Singlet Oxygen Kinetics in E. coli Cells with ACS268 as PS

**Figure 4:**
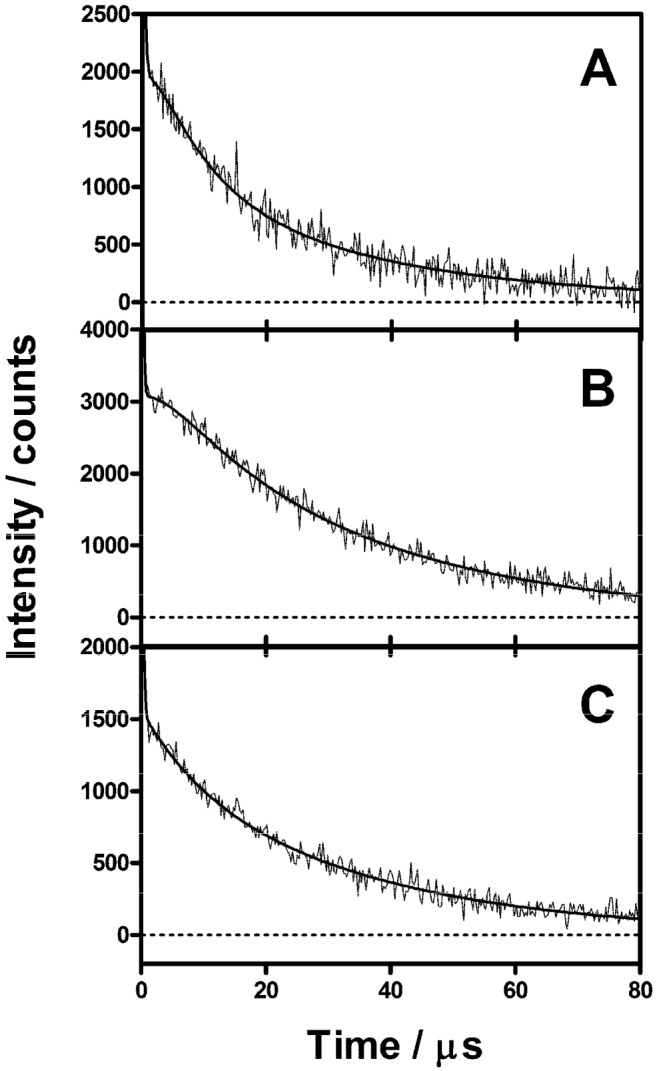
Singlet oxygen phosphorescence at 1270 nm using ACS268 as photosensitizer in (**A**) *E. coli*/PBS in air-saturated atmosphere, (**B**) *E. coli*/D-PBS in air-saturated atmosphere, (**C**) *E. coli*/D-PBS + 0.75 mM BSA in air-saturated atmosphere.

The quantum yield of singlet oxygen production (Φ_Δ_) of ACS268 was measured in two different solvents, DMF and D_2_O, by comparison of the signal produced by optically-matched solutions of reference PSs (phenalenone and TMPyP) [21] with that produced by ACS268. The detailed procedure is described in the Supplementary Information. Thus, Φ_Δ_ values of 0.87 ± 0.01 and 0.04 ± 0.01 were determined in DMF and D_2_O, respectively. Irradiation of ACS268-loaded *E. coli* cells produced time-resolved ^1^O_2_ phosphorescence signals clearly different from those observed for NMB (Figure 4). In PBS, the signals grew with a time constant of τ_1_^PBS^ = 2.1 ± 1 μs, and decayed biexponentially with lifetimes τ_2_^PBS^ = 8.6 ± 1 μs and τ_3_^PBS^ = 34.1 ± 1 μs. In D-PBS, the rise of the signal was slightly slower, τ_1_^D−PBS^ = 5.2 ± 1 μs, but the decay kinetics remained unchanged biexponential. Addition of 0.75 mM of BSA shortened only the slowest decay lifetime, *i.e.*, τ_2_^BSA^ = 8.6 ± 1 μs and τ_3_^BSA^ = 34.0 ± 1 μs. Finally, measurements in *E. coli* spheroplasts led to a 3-fold decrease in the ^1^O_2_ phosphorescence signal intensity, but with the same kinetics as in intact cells (see Supplementary Information).

### 2.5. Discussion

The main goal of this study was to gain insight into the kinetics of ^1^O_2_ production and decay by three different photosensitizers in *E. coli* cells, thereby shedding light on the mechanism of photodynamic inactivation of Gram-negative bacteria. Specifically, we were interested to learn whether representative members of three different families of photosensitizers would provide different photosensitization kinetics.

Towards this goal, it was first essential to obtain unambiguous time-resolved ^1^O_2_ phosphorescence signals from viable cells, *i.e.*, (1) to reduce to a minimum the acquisition time as the ^1^O_2_ kinetics in cells changes with increasing light exposure [22,23], and (2) to identify the optimal concentration and contact time of each PS for efficient cell binding/uptake. A concentration *ca.* 10 μM, similar to those used in inactivation experiments for *E. coli* [15,24], and a 30 min contact time, typical in APDT [25,26], proved adequate in this respect notwithstanding the higher amount of NMB taken up at longer contact times (Figure 2). Under similar experimental conditions, all these PSs show no significant dark toxicity [15,17].

In homogeneous environments, the ^1^O_2_ signals can be appropriately described by Equation (1), where *S_0_* is a quantity proportional to the amount of ^1^O_2_ formed upon pulse excitation, τ_T_ is the lifetime of the PS’s triplet-excited state, and τ_Δ_ is the ^1^O_2_ lifetime. Thus, the signal is expected to show two monoexponential components, corresponding to the rise and decay of the signal:

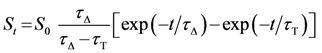
(1)


In microheterogeneous environments it is often found that different populations the PS’s triplet-excited state and ^1^O_2_ exist and therefore Equation (1) must be expanded to account for such different populations Equation (2). In such cases the rise and the decay of the signals follow multiexponential laws, the number of exponentials representing the number of independent populations.

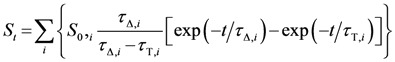
(2)


The kinetic models may be even more complex due to phenomena such as diffusion or cross-talk between the different populations.

Regarding the phenothiazine group, NMB was chosen as the model due to its highest singlet oxygen quantum yield and higher hydrophobicity [27], as well as its ability to properly inactivate many bacterial species [27]. Two major changes can be observed in the absorption spectrum of NMB upon binding to *E. coli*: on one hand, the relative contribution of the shoulder at 600 nm increases, which is consistent with the report by Usacheva *et al.* [28] that phenothiazine dyes tend to dimerize in the presence of bacteria. On the other, the band maximum shows a slight red shift that reveals a change in the NMB’s microenvironment [29]. Somewhat surprisingly, such microenvironmental changes have no effect on the fluorescence spectrum or in the fluorescence lifetime. The fact that there is no change in the fluorescence lifetime indicates that, despite the different microenvironment, NMB is still surrounded by water molecules (see Supplementary Information). This conclusion is supported by the ^1^O_2_ phosphorescence formation and decay kinetics. Both in PBS and D-PBS, the lifetimes obtained for the ^1^O_2_ phosphorescence signal matched those obtained in neat H_2_O or D_2_O, a 2.5 μs rise component assigned to the triplet state of NMB, and a 3.7 or 65 μs component due to ^1^O_2_, respectively. This indicates that NMB is localized in an aqueous-like environment and that ^1^O_2_ is deactivated mainly by interactions with H_2_O or D_2_O [30]. Of course, it cannot be excluded that another fraction of ^1^O_2_ molecules reacts with or is quenched by cell components so rapidly that it does not contribute to the observed signal. Care was taken to ensure that the signals did not originate on NMB molecules that have leaked into the bulk solution during the experiments. The supernatant solution was checked before and after the experiments and in all cases we could find no signal. Addition of BSA to the D_2_O suspensions significantly changed the signal kinetics, causing both a faster rise (<1 μs) and a faster decay (lifetime of 7 μs) in air-saturated suspensions. The latter component was decreased further to 3.5 μs upon bubbling with oxygen, which increased five-fold the concentration of oxygen in the system. These results suggest a dual effect of BSA: on one hand it quenches ^1^O_2_ as one would expect [19] decreasing its lifetime below the time resolution of our setup; on the other, it interacts with NMB shielding it from oxygen [31], which effectively increases the triplet NMB lifetime from 2.5 μs to 7 μs (in air) or 3.5 μs (under oxygen) as the bimolecular energy transfer process is slowed down. Therefore, NMB must be located somewhere in the external structure of the cell wall, accessible to BSA, or must be able to relocate and bind to BSA upon its addition to the solution. This conclusion is consistent with Usacheva’s *et al.* report that phenothiazinium dyes interact with the outer-wall bacterial lipopolysaccharides (LPS) [32,33]. Overall, our results are in line with the report that an initial population of 10^4^ CFU·mL^−1^ of *E. coli* could be totally eliminated by a minimum lethal concentration of 7.8 μM NMB at a light fluence of 3.15 J cm^−2^ [17]. Thus the suggestion that ^1^O_2_ plays an important role in the mechanism of action of NMB [17] is supported by our spectroscopic data.

Concerning the phthalocyanine family, ZnTMPyPz was chosen as a model since Dupouy *et al.* [15] had shown that, using the same uptake conditions as in this work, irradiation with visible light reduced the cell viability by 99.5%. These results can now be rationalized in the light of our findings: First, as observed in the absorption spectrum (Figure 2C), ZnTMPyPz is largely aggregated in *E. coli* as deduced from the broadening of the bands relative to DMF. This aggregation would explain why no signal is observed in the ^1^O_2_ luminescence experiments under conditions similar to those used for the other PSs, even increasing the contact time and the acquisition time. Spesia *et al.* showed that alterations in the cell membrane appear to be the major cause of *E. coli* inactivation upon APDT with visible light and a tetracationic zinc(II) phthalocyanine derivative (ZnPc^4+^) [34]. Assuming that (1) ZnTMPyPz and ZnPc^4+^ localize in a similar microenvironment because of the structural similarity, and that (2) ZnTMPyPz is basically aggregated like in an aqueous media, as observed by the absorption spectrum and the absence of ^1^O_2_ luminescence signal, a localization to the outer structure of the cell wall, comparable to the one obtained with NMB, is tentatively attributed. This conclusion is not contradictory to the 99.5% reduction in cell viability, as there is still a small quantity of monomer, detectable by steady-state (Figure 2D) and time-resolved (Figure S3; see the Supplementary Information) fluorescence measurements, that could be responsible for the production of ROS and the resulting photodynamic effect shown by ZnTMPyPz.

We previously demonstrated that a tetracationic porphyrin such as TMPyP, with the cationic charges peripherally-distributed, localizes in *E. coli* at two different sites: (1) externally bound to the cell wall, probably in a similar localization as NMB and ZnTMPyPz, and (2) inside the cell, bound to nucleic acids [14]. We hypothesize that ACS268, which has a unique cationic charge in the alkyl chain, would localize in a completely different environment than TMPyP, as the cationic charge is far away from the photoactive core and the lipophilicity/hydrophilicity of the molecule is completely different. The totally different Φ_Δ_ values in DMF and D_2_O clearly demonstrate that ACS268 is able to produce ^1^O_2_ only when it is in monomeric form.

Both the spectroscopic measurements and the ^1^O_2_ phosphorescence in *E. coli* are in agreement with the aforementioned hypothesis. The red shift observed in the absorption and fluorescence spectra in *E. coli* indicates a different environment surrounding the PS relative to that in solution [29,35,36].

With ACS268, two components with lifetimes 8.6 μs and 34 μs are observed in the ^1^O_2_ phosphorescence experiments in PBS, D-PBS and upon addition of BSA. The presence of two lifetimes suggests two different localizations for ACS268 in *E. coli*., likely in the cell wall as deduced from the 3-fold decrease in the ^1^O_2_ phosphorescence signal intensity observed in spheroplasts. Both ACS268 populations are clearly shielded from oxygen as evidenced by the long triplet lifetimes as compared to the 2.5 μs found for NMB. This in turn indicates that only a minor fraction of triplets is quenched by oxygen and therefore that the amount of ^1^O_2_ produced by ACS269 in the cells must be very low. Moreover, the τ_Δ_ values for ^1^O_2_ in *E. coli* are significantly shorter (2.1 μs in PBS; 5.2 μs in D-PBS suspensions) than the ones observed in buffer (3.5 μs in PBS; 67 μs in D-PBS) [30] indicating a fast deactivation of ^1^O_2_ by quenchers surrounding the PS molecules that, however, does not lead to cell death. The inefficient production of singlet oxygen and its fast deactivation are consistent with the very low photodynamic activity found for this compound against *E. coli* (this work, data not shown).

The rate constant of ^1^O_2_ decay in microheterogeneous systems under exchange equilibrium conditions, *k*_d_, is described by Equation (3) [37]:

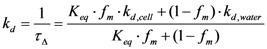
(3)
where *K_eq_* = [^1^O_2_]_cell_/[^1^O_2_]_water_ is the partition equilibrium of ^1^O_2_ between the two phases, *f_m_* and (1-*f_m_*) are the volume fractions of the cell and aqueous phase, respectively, and *k_d,cell_* and *k_d,water_* are the decay rate constants inside and outside the cell, respectively. Assuming *K_eq_* ≈ 1 and *f_m_* ≈ 0.0012 for our suspensions in 5 × 10^8^ CFU/mL, the lifetime of ^1^O_2_ within the bacterial cell can be estimated as τ_Δ,cell_ ≈ 7 ns, a value that would perfectly correlate with the ^1^O_2_ phosphorescence kinetics observed upon addition of BSA, which traps any ^1^O_2_ molecule escaping from the cell wall into the external aqueous media.

It has previously been demonstrated that the presence of external proteins clearly modifies the effectiveness of APDT to inactivate microbial cells [38,39,40]. Such effect can also be observed in the kinetics of ^1^O_2_ in the presence of BSA. The addition of BSA clearly modifies the ^1^O_2_ phosphorescence signal produced by NMB, decreasing τ_Δ_ and increasing τ_T_, demonstrating an interaction between NMB and BSA (Figure 3). However, the effect is barely observed when ACS268 is used as the PS (Figure 4), suggesting a deeper localization of ACS268 into the cell wall, thereby making it less accessible to BSA.

## 3. Experimental

### 3.1. Chemicals

New Methylene Blue N (NMB) was supplied by Sigma-Aldrich Co. (St. Louis, MO, USA). Zinc-tetramethyltetrapyridino[3,4-b:39,49-g:30,40-l:3-,4-q]porphyrazinium salt (ZnTMPyPz) was synthesized as described by Marti *et al.* [20]. 5,10,15-tris(2,6-Dichlorophenyl)-20-[*N*-(12-trimethylammonium chloride)-dodecyl-*p*-sulphonamidophenyl]porphyrin (ACS268) was synthesized as described in the Supplementary Information. Deuterium oxide (D_2_O, >99.9%) and dimethylformamide were purchased from Solvents Documentation Synthesis (Peypin, France). Bovine serum albumin (98%), lysozyme, sodium dodecyl sulphate (SDS), trizma, and Dubelcco’s phosphate buffered saline (PBS) were purchased from Sigma-Aldrich Co. Saccarose and ethylendiamine tetraacetic acid (EDTA) were supplied by Panreac S.A. (Barcelona, Spain). Deuterated PBS (D-PBS) was prepared by dissolving PBS powder in D_2_O instead of H_2_O.

### 3.2. Bacterial Growth

*Escherichia coli* CECT101 was grown overnight in Luria-Bertani (LB) media, then transferred into new LB media to obtain an initial optical density value of 0.1 per cm at 660 nm, and allowed to grow aerobically at 37 °C. Cells in the middle logarithmic phase (optical density 0.35 per cm at 660 nm) were harvested by centrifugation (10 min, 3,000 rpm) and then resuspended in sterile PBS at pH 7.4. The procedure was repeated three times, yielding a bacterial concentration of 10^8^ colony forming units (CFU) mL^−1^, as assessed by serial dilutions of the cell suspension followed by a colony formation assay.

### 3.3. PS Binding to E. coli

The bacterial uptake of PS was determined by fluorescence spectroscopy following the procedure described by Hamblin *et al.* [26,41]. Bacterial suspensions (10^8^ CFU·mL^−1^) were incubated in the dark at room temperature (NMB, ACS268) or at 37 °C (ZnTMPyPz) with varying concentrations of PS and contact times under gentle stirring. Afterwards, the cells were washed by centrifugation (10 min, 3,000 rpm, 3 times) to remove any excess of PS. After the last centrifugation, a solution of 1% SDS in 0.1 M NaOH was added to the pellets and shaken for a minimum of 24 h. Each experiment was repeated three times. Stock PS solutions were sterilized by filtration through a 0.22 µm sterile filter and stored in the dark at 4 °C for a maximum of 1 week.

### 3.4. Spheroplasts Formation

Spheroplasts were obtained by resuspending treated or untreated *E. coli* cells in Tris-HCl 0.05 M buffer at pH = 6.8 containing 0.01 M EDTA, 0.3 M sucrose and lysozyme (1 mg mL^−1^) under stirring. After 1 h incubation at 37 °C, tubes were centrifuged twice at 2,000 rpm for 10 min [42]. The pellet was then resuspended in PBS solution at pH = 7.4. PS-loaded spheroplasts were obtained by first incubating whole *E. coli* cells with the PS and then subjecting them to the procedure above.

### 3.5. General Spectroscopic Measurements

Absorption spectra were recorded on a Cary 4E spectrophotometer (Varian, Palo Alto, CA, USA), equipped with a 110 mm diameter integrating sphere and high performance photomultiplier tube for transmittance measurements. Fluorescence emission spectra were recorded in a Spex Fluoromax-2 spectrofluorometer (Horiba Jobin-Yvon, Edison, NJ, USA). Fluorescence decays were recorded with a time-correlated single photon counting system (Fluotime 200, PicoQuant GmbH, Berlin, Germany) equipped with a red sensitive photomultiplier. Excitation was achieved by means of a 375 nm picosecond diode laser, or a 596 or 657 nm LED working at 10 MHz repetition rate. The counting frequency was always below 1%. Fluorescence decays were analysed using the PicoQuant FluoFit 4.0 data analysis software.

### 3.6. Time-Resolved Singlet Oxygen Measurements

^1^O_2_ phosphorescence detected by means of a customized PicoQuant Fluotime 200 system described in detail elsewhere [43]. Briefly, a diode-pumped pulsed Nd:Yag laser (FTSS355-Q, Crystal Laser, Berlin, Germany) working at 10 kHz repetition rate at 532 nm (12 mW, 1.2 μJ per pulse) was used for excitation. A 1,064 nm rugate notch filter (Edmund Optics, Barrington, NJ, USA) was placed at the exit port of the laser to remove any residual component of its fundamental emission in the near-IR region. The luminescence exiting from the side of the sample was filtered by a cold mirror (CVI Melles Griot, Albuquerque, NM, USA) to remove any scattered laser radiation, and focused on the entrance slit of a Science Tech 9055 dual grating monochromator. A near-IR sensitive photomultiplier tube assembly (H9170-45, Hamamatsu Photonics, Hamamatsu City, Japan) was used as detector at the exit port of the monochromator. Photon counting was achieved with a multichannel scaler (Becker&Hickl MSA 300 or PicoQuant’s Nanoharp 250). The system is similar to that used in other laboratories [44]. Time-resolved ^1^O_2_ signals were analysed by fitting either Equation (1) or (2) to the data using the FluoFit software. The lifetime values quoted throughout the manuscript refer to mean and standard deviation values derived from at least three independent measurements. All spectroscopic measurements were carried out in 1-cm quartz cuvettes (Hellma, Müllheim, Germany) at room temperature.

### 3.7. Measurements in Bacterial Suspensions

In a typical experiment, bacterial suspensions were incubated in the dark with the desired amount of PS for a given period of time. The cells were then washed three times with PBS and resuspended in PBS to a final concentration of ~5 × 10^8^ CFU·mL^−1^. For time-resolved phosphorescence measurements, the bacterial suspensions (3 mL) were irradiated with 3 million laser pulses at 532 nm. The suspensions were gently stirred during the measurements. Appropriate controls were performed to ensure that the signals originated from PS molecules bound to the bacterial cells (see Supplementary Information).

## 4. Conclusions

In summary, we have demonstrated that, between the different families used in the study, there are remarkable differences in (1) the microenvironment surrounding the PSs, *i.e.*, in their localization, and (2) the kinetics of production and decay of ^1^O_2_. When bound to *E. coli*, NMB localizes in an aqueous-like microenvironment, accessible to external proteins such as BSA and interacting with the LPS in cell-wall, ZnTMPyPz might localize similarly to NMB, where it is almost completely aggregated, and ACS268 localizes in a deeper position of the external structure of the cell wall, namely in the outer membrane, shielded from oxygen as demonstrated with the triplet lifetimes. With regards to the initial location of ^1^O_2_ produced by the PSs, ^1^O_2_ generated by NMB can move freely within the cell being mainly deactivated by the aqueous phase, while a completely different scenario is observed for ACS268, suggesting that ^1^O_2_ cannot escape freely from its primary site of production, and thus partially reacts with proximate cellular components. On the other hand, ZnTMPyPz did not show ^1^O_2_-luminescence under conditions similar to those used for the others PSs, correlating with the lower photodynamic inactivation effect observed. Finally, the correlation between the spectroscopic results and the antimicrobial activity shown by these different photosensitizers indicates that the time-resolved monitoring of ^1^O_2_ phosphorescence is a convenient tool to assess the efficiency of antimicrobial photodynamic treatments.
